# Gastrointestinal Bleeding in Cirrhotic Patients with Portal Hypertension

**DOI:** 10.1155/2013/541836

**Published:** 2013-07-22

**Authors:** Erwin Biecker

**Affiliations:** Department of Gastroenterology and Hepatology, HELIOS Klinikum Siegburg, Ringstraße 49, 53129 Siegburg, Germany

## Abstract

Gastrointestinal bleeding related to portal hypertension is a serious complication in patients with liver cirrhosis. Most patients bleed from esophageal or gastric varices, but bleeding from ectopic varices or portal hypertensive gastropathy is also possible. The management of acute bleeding has changed over the last years. Patients are managed with a combination of endoscopic and pharmacologic treatment. The endoscopic treatment of choice for esophageal variceal bleeding is variceal band ligation. Bleeding from gastric varices is treated by injection with cyanoacrylate. Treatment with vasoactive drugs as well as antibiotic treatment is started before or at the time point of endoscopy. The first-line treatment for primary prophylaxis of esophageal variceal bleeding is nonselective beta blockers. Pharmacologic therapy is recommended for most patients; band ligation is an alternative in patients with contraindications for or intolerability of beta blockers. Treatment options for secondary prophylaxis include variceal band ligation, beta blockers, a combination of nitrates and beta blockers, and combination of band ligation and pharmacologic treatment. A clear superiority of one treatment over the other has not been shown. Bleeding from portal hypertensive gastropathy or ectopic varices is less common. Treatment options include beta blocker therapy, injection therapy, and interventional radiology.

## 1. Introduction

One of the main complications of liver cirrhosis is portal hypertension. Portal hypertension is defined as an hepatic venous pressure gradient (HVPG) above 5 mmHg. Clinical significant complications of portal hypertension like development of ascites and/or esophageal and gastric varices usually develop at an HVPG above 10 mmHg [1]. Bleeding from esophageal or gastric varices still carries a significant morbidity and mortality risk. The prevention of a first bleeding episode and the management of acute bleeding have markedly improved over the last years. This paper gives a concise overview of the current recommendations for the prevention and treatment of bleeding from esophageal, gastric, and ectopic varices as well as bleeding from portal hypertensive gastropathy. 

## 2. Natural History of Esophageal Varices

At the first diagnosis, about 30 to 40% of patients with compensated cirrhosis of the liver and 60% of patients with ascites present with esophageal varices. The annual incidence for the development of new varices in patients who were diagnosed with liver cirrhosis without varices is between 5 and 10% [2–5]. Once varices have developed, they have the tendency to increase in size and may eventually rupture and bleed [3]. Variceal hemorrhage still carries a significant mortality of 7%–15% [6–8]. 

Risk factors for variceal bleeding are the diameter of the varix, presence of red wale signs, and an impaired liver function [9–12]. Hemodynamic studies point at a close association of the HVPG and the bleeding risk [11].

## 3. Primary Prophylaxis of Esophageal Variceal Bleeding

**Figure 1:**
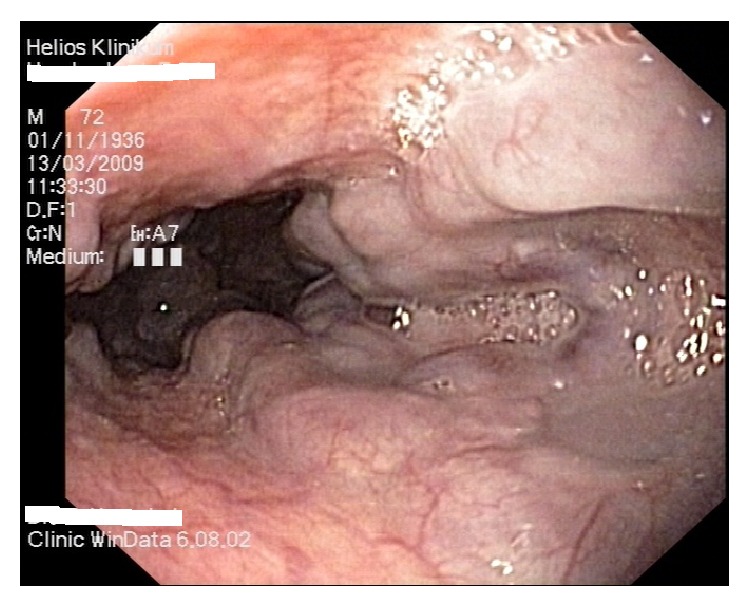
Esophageal varices in a patient with liver cirrhosis, second grade.

The identification and prophylactic treatment of patients at risk of esophageal variceal bleeding is a major goal [13]. Every patient with newly diagnosed liver cirrhosis should be screened for esophageal and/or gastric varices [13]. In patients with esophageal varices (Figure 1) at risk of bleeding, prophylactic treatment should be initiated, since the risk of bleeding is 30–35% in two years [14]. Patients at high risk of bleeding are those with medium or large varices and small varices with red wale signs and patients in a Child class C state. Effective prophylactic treatment reduces the risk of bleeding by about 50% [15].

Prophylactic treatment is not necessary when the bleeding risk is low. There is no effective prevention for the development of gastroesophageal varices in patients with cirrhosis [1]. Only one study has shown that prophylaxis with a nonselective beta blocker is effective in preventing the enlargement of small varices [16]. Nevertheless, endoscopic follow-up is mandatory [13]. Esophageal varices tend to increase in size in a linear fashion. One study including 258 patients with small varices and without a history of variceal bleeding found an increase in variceal size in 21%, 45%, and 66% of the patients after 1.5, 3, and 4.5 years, respectively [17]. However, the course of the underlying liver disease is a major determinant of variceal progression [9, 17]. The actual recommendation for surveillance in patients with compensated liver disease and small varices at the initial endoscopy is a follow-up examination after 1-2 years [13, 18]. If the initial endoscopy has shown no varices, a follow-up examination after 2-3 years is sufficient in patients with compensated liver disease [13, 17, 18]. Patients with decompensated liver disease should have shorter surveillance intervals.

Therapy options for primary prophylaxis include nonselective beta blockers and endoscopic variceal band ligation (VBL). Endoscopic sclerotherapy and shunt procedures are nowadays obsolete in primary prophylaxis.

Nonselective beta blockers cause vasoconstriction of the splanchnic circulation by *β*_2_-receptor inhibition and decrease cardiac output by *β*_1_-receptor blockade. This results in unopposed alpha-1 activity, leads to a decrease in portal venous inflow, and thereby lowers portal pressure.

Not all patients receiving beta blockers respond with a reduction of the HVPG [19]. Clinical studies have shown that at most 50% of beta blocker treated patients achieved a reduction of the HVPG below 12 mmHg or >20% from baseline levels [19]. However, other effects of beta blocker therapy besides the reduction of HVPG like a decrease in azygos blood flow or a decrease in bacterial translocation from the gut [20] may play a role in the prevention of variceal hemorrhage [21].

Nonselective beta blockers like propranolol and nadolol were introduced for primary prophylaxis almost 30 years ago [15]. In recent years, carvedilol was shown to be effective, too. Carvedilol is a noncardioselective vasodilating beta blocker with mild intrinsic anti-*α*(1)-adrenergic activity. Carvedilol is at least as effective in lowering HVPG as propranolol [22] or nadolol plus nitrate [23] and is as effective as VBL for primary prophylaxis of variceal bleeding [24]. Monotherapy with nitrates or a combination of beta blockers and nitrates compared to beta blockers alone has no benefit in primary prophylaxis [15, 25]. Meta-analysis has shown a reduction of the bleeding risk by a nonselective beta blocker of about 50%. Around 20% of patients suffer from intolerable side effects that require discontinuation of the drug. After discontinuation, the bleeding risk is not different from an untreated population. That makes an indefinite prophylactic therapy necessary [26]. The most important predictor for variceal bleeding in patients on a therapy with beta blockers is the dose of the drug [27]. Patients should therefore receive the highest tolerated dose.

**Figure 2:**
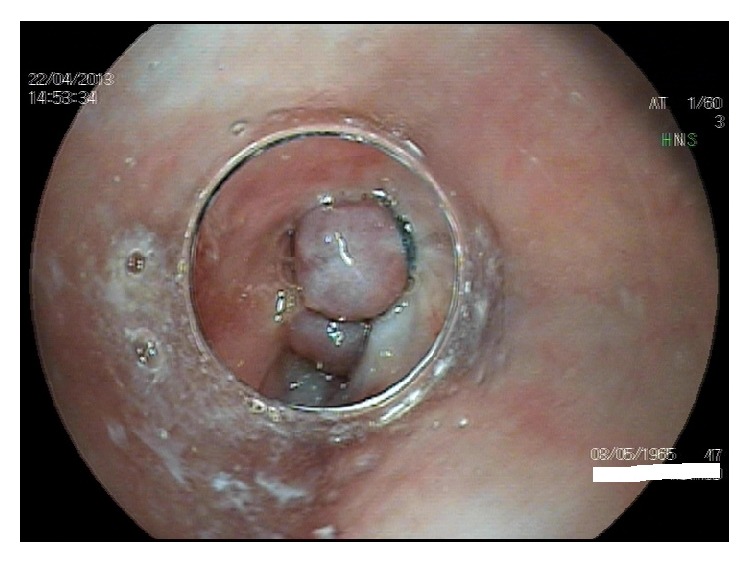
Endoscopic view through the transparent cap mounted at the tip of the endoscope at a ligated varix in the lower esophagus.

An effective alternative option for primary prophylaxis is endoscopic VBL [28–31] (Figure 2). Compared to untreated controls, prophylactic VBL reduces the risks of variceal bleeding and mortality [32]. Several studies compared endoscopic VBL with propranolol for primary prophylaxis of variceal bleeding [28–31]. Three studies found no difference between beta blockers and VBL concerning prophylaxis of bleeding [28, 30, 31]. One study that is controversially discussed because of some methodological flaws found a significant benefit for endoscopic VBL [30]. A recently published Cochrane analysis that included 19 randomized trials found a slight beneficial effect for VBL, but that effect was not present when only full-published paper articles were analyzed [33]. In terms of efficacy, VBL and nonselective beta blocker therapy are considered to be equivalent. 

Because of the low costs, ease of administration with no need of special endoscopic expertise, and the absence of procedure-related mortality, nonselective beta blockers are recommended as first-line treatment for the primary prophylaxis of esophageal variceal bleeding [15].

**Figure 3:**
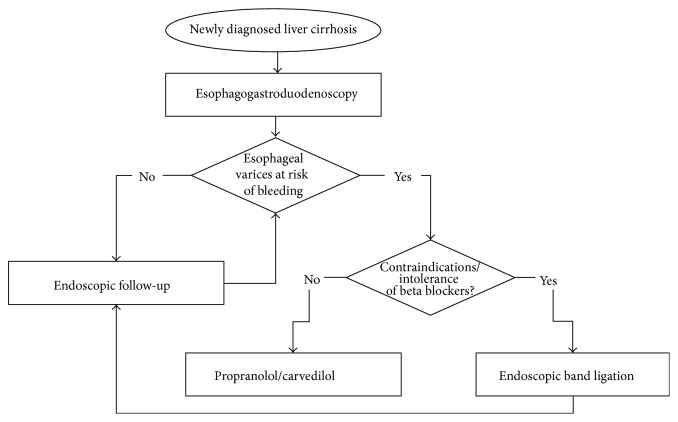
Flow chart showing the options for primary prevention of esophageal variceal bleeding. Patients at high risk of bleeding are those with medium or large varices, small varices, and red whale signs and patients in a Child class C state.

Variceal band ligation is recommended in patients with serious side effects or intolerability of beta blocker therapy as well as in patients with contraindications for drug therapy.  Figure 3 shows a flow chart for the prophylactic treatment of esophageal varices.

## 4. Acute Bleeding from Esophageal Varices

More than two-thirds of all bleeding episodes in patients with liver cirrhosis are caused by esophageal variceal hemorrhage [34]. Hence, every patient with liver cirrhosis and signs of gastrointestinal bleeding should be treated as having variceal hemorrhage until a definite diagnosis is made.

About 40–50% of variceal hemorrhages cease spontaneously without therapeutic intervention [14]. Control of acute bleeding is achieved in more than 80% of patients using actual therapy modalities. In the remaining 20% of patients either control of acute bleeding is not possible or recurrence of the bleeding in the first six weeks after the initial bleeding episode, with the highest risk in the first five days, will occur [34–36]. Six weeks after the bleeding episode the risk of bleeding equals the initial risk before the bleeding [37]. Rebleeding within the first five days is associated with a high mortality and was analyzed in most studies as a composite endpoint referred to as 5d failure. Risk factors of early mortality include Child-Pugh class and MELD score [6, 34, 38], infection [38, 39], active bleeding on admission [38, 39], portal vein thrombosis [34], and a high initial HVPG (>=20 mmHg) [6, 40, 41]. Over the last 25 years the mortality associated with acute esophageal variceal bleeding has decreased from more than 40% to 15 to 20% [7, 34, 36, 42, 43].

Acute bleeding from esophageal varices is a medical emergency and often a dramatic event. Most patients vomit blood, but hematochezia and melena might be the only symptoms. Dependent on the amount of lost blood, patients might be hemodynamic instable and present in hemorrhagic shock. Patients should be managed on an intensive care unit by a multidisciplinary team consisting of endoscopists, hepatologists, and specially trained nurses as well as interventional radiologists and surgeons if necessary. Today only 40% of patients die from exsanguinating bleeding. The outcome of patients is directly correlated to the extent of organ failure. Most deaths are caused by complications of bleeding like liver failure, infections, and hepatorenal syndrome [34, 36]. Risk factors for an adverse course are the degree of liver dysfunction, creatinine, hypovolemic shock, active bleeding on endoscopy, and presence of hepatocellular carcinoma [7, 34, 36, 37, 39, 44]. Thus, the management of patients with acute variceal bleeding includes not only treatment and control of active bleeding but also the prevention of rebleeding, infections, and renal failure [45].

 If variceal bleeding is suspected, patients should be hemodynamically stabilized and receive medical treatment with vasopressors and antibiotic therapy [46–50]. In uncomplicated patients antibiotic therapy is done using quinolones [51]. High-risk patients with advanced liver disease (ascites, encephalopathy, jaundice, and malnutrition) or previous treatment with quinolones should receive ceftriaxone [48]. Antibiotic therapy of patients with acute variceal bleeding does not only decrease mortality but also decrease the probability of rebleeding [49]. Transfusion of blood should be done with caution with a target hemoglobin level between 7-8 g/dL, since higher hemoglobin levels can increase portal pressure [52], and restrictive transfusion strategies are associated with better survival [53]. Patients with variceal bleeding are at high risk of bronchial aspiration. Extreme care of the airways is therefore mandatory. Patients with massive bleeding and/or patients who present with hepatic encephalopathy should undergo endotracheal intubation and mechanical ventilation prior endoscopy to prevent aspiration pneumonia [54]. Another concern in cirrhotic patients is the often impaired blood coagulation due to a low platelet count and impaired plasmatic coagulation. These factors have long been considered as risk factors in variceal hemorrhage, but in reality these factors are only poorly correlated with variceal bleeding [55].

**Figure 4:**
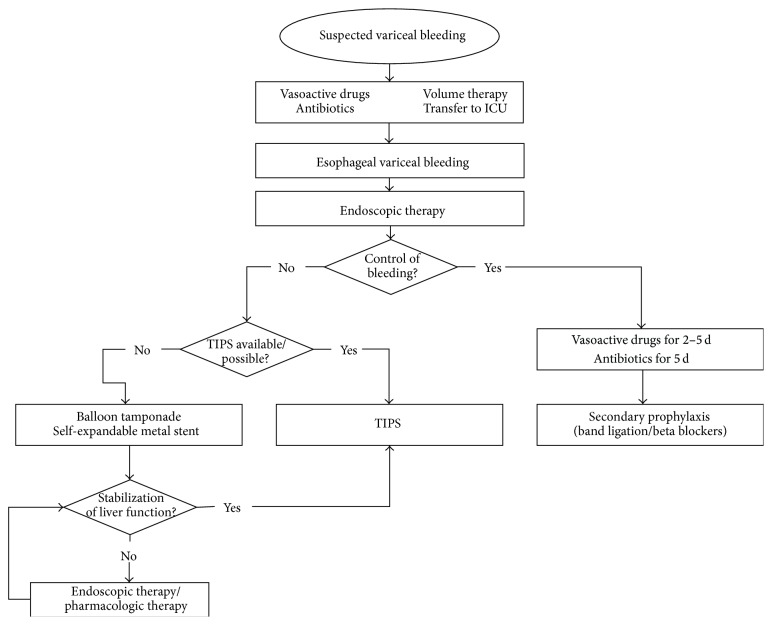
Flow chart showing the management of acute bleeding from esophageal varices. As an option in high-risk patients, early insertion of a PTFE covered TIPS is an alternative to endoscopic and vasopressor treatment.

Available therapy options include pharmacologic and endoscopic treatment, balloon tamponade, placement of fully covered self-expandable metallic stents, transjugular intrahepatic shunt (TIPS), and surgical shunts. Nowadays, the initial approach is a combination of vasoactive drugs, antibiotics, and endoscopic therapy [56].  Figure 4 shows a treatment algorithm for acute esophageal variceal bleeding.

### 4.1. Pharmacological Therapy

The aim of pharmacological therapy is to reduce splanchnic blood flow and portal pressure and thereby control of active bleeding [57, 58]. Hemostasis is achieved in more than 80% of patients using vasoactive drugs alone [59]. Vasoactive drugs currently in use are vasopressin, somatostatin, and, most important in Europe, terlipressin. Vasoactive drugs should be given whenever variceal bleeding is suspected, ideally as soon as possible, as about 25% of patients die very early after onset of bleeding [60]. Moreover, active bleeding during endoscopy is reduced when vasoactive drugs are given before endoscopy, and thereby the success rate of endoscopic therapeutic procedures is higher [61–63]. 

Vasopressin is the most potent splanchnic vasoconstrictor. Due to its short half-life, vasopressin has to be given as a continuous i.v. infusion. Relevant adverse effects include systemic vasoconstriction with serious implications like mesenteric or myocardial ischemia [64]. Application of vasopressin in combination with nitrates reduces the side effects associated with vasoconstriction [65, 66]. Several studies have shown that treatment with vasopressin is effective in terms of bleeding control but does not affect mortality [64, 67–69]. Terlipressin is a synthetic vasopressin analogue with a longer half-life and less adverse effects. Its effect is still significant 4 hours after administration [70–72]. Several studies have shown that terlipressin is effective in bleeding control and has a positive impact on survival [61, 73–75]. Terlipressin achieves control of bleeding in 75–80% and 67% of patients at 48 hours and at five days, respectively [75, 76]. It is given at a dose of 2 mg every 4 hours for the first 48 hours and could be continued for prevention of early rebleeding at a dose of 1 mg every 4 hours for up to 5 days [72, 76]. A recent study has shown a drop of serum sodium in the range of >5 mEq/L in 67% of patients and of >10 mEq/L in 36% of patients treated with terlipressin [77]. Therefore, serum sodium should be monitored in patients receiving terlipressin. Compared to vasopressin, terlipressin is more effective in control of esophageal bleeding [73, 78], and compared to vasopressin plus nitrate [79] as well as compared to somatostatin it is comparably effective [80, 81]. Terlipressin has to be used with caution in patients with ischemic heart disease and in patients with peripheral vascular disease as it may cause ischemic complications as well as relevant dysrhythmias.

Somatostatin causes splanchnic vasoconstriction as well as a reduction in portal pressure and HVPG [82–84]. Furthermore, it inhibits the postprandial increase in portal blood flow and portal pressure. 

The efficacy of somatostatin in variceal bleeding was shown in several trials. Compared to placebo, control of bleeding is achieved in 63% versus 46%, respectively [62, 84, 85], but a benefit in survival was not shown [14]. 

Somatostatin is given as an initial bolus of 250 *μ*g followed by a 250 to 500 *μ*g/h continuous infusion until a bleed-free period of 24 hours is achieved [45]. The effect on HVPG seems to be dose related [82], and higher doses might therefore be more effective in severe bleeding [86]. Somatostatin is as effective as vasopressin in control of variceal bleeding; the safety profile is superior to vasopressin [87].

Octreotide is a synthetic analogue of somatostatin with a longer half-life. The longer half-life, however, is not reflected by longer hemodynamic effects [88, 89], what might be caused by rapid desensitization or tachyphylaxis [90]. Since convincing studies are lacking [91], octreotide is not recommended as a single therapy in acute variceal hemorrhage. The combination of terlipressin and octreotide is not superior to a monotherapy with terlipressin [92]. One meta-analysis has shown that octreotide is effective in the prevention of rebleeding when it is given in addition to endoscopic therapy [93]. Octreotide is administered as an initial bolus of 25 *μ*g, followed by an infusion of 25 to 50 *μ*g/h [94]. Both somatostatin and octreotide have a good safety profile. Possible adverse effects include mild hyperglycemia and abdominal cramps. 

In summary, the available data is most convincing for terlipressin; however, the direct comparison of terlipressin and octreotide revealed no superiority of terlipressin [91, 95].

### 4.2. Endoscopic Therapy

About 80–90% of acute variceal bleeding episodes are successfully controlled by endoscopic therapy [96, 97]. Nowadays, most important is endoscopic VBL. Injection therapy using sclerosing agents like ethoxysklerol or cyanoacrylate is less commonly used. Aethoxysklerol is injected next to—not into—the varix. It causes local inflammation and scaring and thereby thrombosis and obliteration of the vessel. On the other hand, cyanoacrylate is injected directly into the varix, causing immediate obliteration of the vessel. Endoscopic band ligation is done using a transparent cap that is attached to the tip of the endoscope. By applying suction, the varix is then pulled into the cap and a rubber ring is thrown over the varix causing thrombosis and scaring of the vessel. 

Before the introduction of VBL, aethoxysklerol injection was widely used in the treatment of acute esophageal variceal bleeding. Studies have shown that sclerotherapy was at least as effective as balloon tamponade [98, 99]. The injection of cyanoacrylate is used as a second-line therapy when VBL of variceal bleeding fails.

Endoscopic VBL was first carried out in 1988 [100]. The method is now widely available, and complications are—compared to sclerotherapy—less common [101]. The most frequent complications are superficial ulcerations and esophageal strictures. Bleeding after the rubber rings have fallen off is less common. A disadvantage of the method is the impaired sight that is caused by the ligation system. Costs are—compared to sclerotherapy—higher. Mortality rates after VBL are lower as compared to sclerotherapy [102, 103].

### 4.3. Combination Therapy

Several randomized controlled trials as well as meta-analyses of these trials have shown that the combination of endoscopic treatment and vasoactive drugs is superior to either treatment alone. This was shown for a combination of endoscopic sclerotherapy with vasoactive drugs [56] and for a combination of VBL and octreotide with a significant reduction in early rebleeding (VBL alone 38% versus 9% for the combination therapy) and a trend for the reduction in 30 d mortality (VBL 23% versus 11% for the combination therapy) [104]. Another trial using combination therapy of endoscopic treatment (either sclerotherapy or VBL) and pharmacologic treatment with vapreotide (a somatostatin analogue) found the combination treatment to be superior to endoscopic treatment alone in the control of bleeding, but a difference in mortality was not evident [63]. However, when pooling the patients from the above-mentioned studies who underwent VBL, a reduction in early mortality is evident [54]. Only one full-published paper compared combination therapy (sclerotherapy and somatostatin) with somatostatin treatment alone [105]. The authors found combination therapy more effective than somatostatin alone with therapeutic failure occurring in 21 patients of the somatostatin group and in 7 patients of the combined therapy group. Failure to control the acute episode occurred in 24% versus 8% and early rebleeding in 24% versus 7%, respectively [105]. One trial compared VBL in combination with terlipressin versus terlipressin alone in patients with no active bleeding on endoscopy. The combination therapy was superior to a monotherapy with terlipressin in the reduction of early rebleeding (0% versus 15%) and treatment failure (2% versus 24%) [106]. 

In summary, the combination of endoscopic VBL and vasoactive drugs (preferably terlipressin) is clearly more effective in the control of active bleeding and in the prevention of rebleeding than endoscopic or pharmacologic treatment alone. The effect on mortality was less clearly shown in the available studies, but this might be related to an insufficient number of patients included in the studies.

### 4.4. Balloon Tamponade

The use of balloon tamponade for the treatment of acute esophageal variceal bleeding was introduced by Sengstaken and Blakemore in 1950 [107]. The Minnesota tube is a modified version with an aspiration channel above the esophageal balloon. For uncontrolled bleeding from gastric varices, the Linton-Nachlas tube is preferred [108]. In the hand of the experienced user the method allows control of bleeding in most patients [109]. A major drawback of the method is the high amount of possible serious complications like necrosis and/or rupture of the esophagus as well as aspiration pneumonia [110]. Deflating of the balloon after six hours reduces the risk of complications. Due to the serious risks, balloon tamponade should only be applied by an experienced physician under fluoroscopic control. After all, balloon tamponade is only a bridging procedure until other definite therapy options are available.

### 4.5. Self-Expandable Metal Stents

The placement of fully covered self-expandable metal stents (SEMS) is an alternative to balloon tamponade. The SEMS is inserted over an endoscopic placed guide wire using a stent delivery device without the need of fluoroscopy [111]. SEMS controls bleeding by compression of the bleeding varices [111]. The stent can be left in place for up to two weeks and can be easily removed by endoscopy. The effectiveness in the control of refractory esophageal variceal bleeding has been shown in four case series [112–115]. The procedure is safe with minor complications like esophageal ulcerations, compression of the bronchial system, and stent migration into the stomach being described [112–115]. Like balloon tamponade, the procedure is reserved for patients with bleeding refractory to medical and endoscopic treatment. It does not allow definite treatment of variceal bleeding due to the high percentage of patients with rebleeding after the SEMS has been removed but has to be considered as an effective and safe bridging procedure that allows stabilization of the patient until definite treatment is possible.

### 4.6. Transjugular Intrahepatic Portosystemic Shunt (TIPS)

By TIPS placement a functional portacaval side-to-side shunt is established. TIPS is indicated in patients with refractory acute variceal bleeding that could not be sufficiently controlled by endoscopic and/or medical therapy and in patients with recurrent bleeding despite optimal endoscopic therapy. After TIPS insertion, bleeding is stopped in almost all of the affected patients [116–118]. The rate of recurrent bleeding after one year is 8–18% [119–121]. However, TIPS insertion is a problem in patients with multiorgan failure and/or in patients with decompensated liver disease. In these patients, the 30-day mortality rate is as high as 100% [116–118, 122]. Disadvantages of the procedure are the risk of hepatic encephalopathy as well as TIPS dysfunction with the risk of recurrent bleeding [123, 124]. A major improvement was the introduction of polytetrafluoroethylene (PTFE) covered stents. These stents have higher rates of patency over time, and mortality rates are lower [125]. A recently published trial has investigated the role of early TIPS in high-risk patients [126, 127]. The multicenter study including 63 patients with esophageal hemorrhage and a high risk of treatment failure (Child B with active bleeding or Child C < 14 points) demonstrated that insertion of a PTFE covered TIPS within 72 h (preferable within 24 h) compared to combined endoscopic and vasoactive drug treatment decreased rebleeding (50% patients without rebleeding in the non-TIPS versus 97% without rebleeding in the TIPS group) and 1-year mortality (86% survival in the TIPS versus 61% survival in the non-TIPS group) [126]. These results are very promising and make the early insertion of a PTFE covered TIPS in high-risk patients an alternative to the combination of pharmacologic and endoscopic treatment. 

### 4.7. Surgery

Surgical procedures in patients with acute or recurrent variceal bleeding are limited to a very small portion of patients in whom medical and/or endoscopic control of bleeding was not achievable, and TIPS was not an option because of technical or anatomical problems (e.g., complete thrombosis of the portal vein). Possible procedures are portosystemic shunt operations [128] or staple transection of the esophagus [129]. Survival of patients who have undergone surgery is dependent on liver function, but the mortality rate is as high as 80%.

## 5. Secondary Prophylaxis of Esophageal Variceal Bleeding

**Figure 5:**
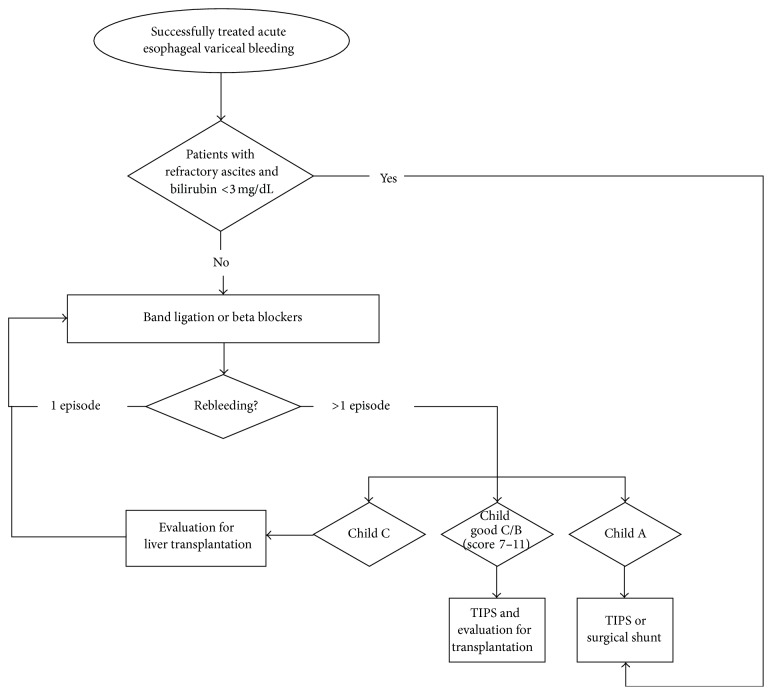
Flow chart giving the possible options for patients with rebleeding from esophageal varices (secondary prophylaxis).

In patients who survive the first episode of esophageal variceal hemorrhage, the risk of recurrent bleeding is as high as 60% with a mortality rate of up to 33% [130]. Prevention of rebleeding is therefore a major goal in patients in whom the initial bleeding episode has been successfully controlled.  Figure 5 shows an algorithm for the secondary prophylaxis of esophageal variceal bleeding.

### 5.1. Pharmacological Therapy

Several studies are available that compared the nonselective beta blockers propranolol or nadolol with no prophylaxis after initial bleeding [131–139]. Most of the studies found a reduction of the rebleeding risk as well as a reduction in mortality. Addition of nitrates further increased this positive effect [140]. Essential is a reduction of the HVPG of at least 20%, even if a reduction below 12 mmHg could not be achieved [27, 141–143]. 

### 5.2. Endoscopic Therapy

Several groups studied the effect of sclerotherapy for secondary prophylaxis of variceal bleeding [137, 144–148]. The comparison of sclerotherapy to medical therapy with a nonselective beta blocker found a benefit for patients treated with sclerotherapy in two studies [149, 150] and a slight but statistically not significant benefit for beta blocker therapy [137, 151, 152]. Three more studies did not find a difference between the two treatment modalities [149, 150, 153].

For prophylaxis of recurrent bleeding, sclerotherapy is now replaced widely by VBL. Several studies have shown the superiority of VBL over sclerotherapy [101, 103, 154–158].

Comparing VBL to medical therapy with nonselective beta blockers in combination with nitrates, two studies found medical therapy to be as effective [142] as or more effective [159] than VBL. In contrast, one study found VBL to be advantageous over medical therapy [160]. From the pathophysiological point of view, the combination of VBL and medical therapy is an even more promising approach for secondary prophylaxis. This has been investigated in five studies [106, 161–164]. Whereas two studies found combination therapy to be more effective than VBL alone [161, 164], two more recent studies that compared nadolol plus nitrates with the combination treatment of drugs and VBL, failed to demonstrate superiority of combination treatment [106, 162]. Therefore, it seems that a clear recommendation of medial treatment alone, VBL alone, or combination treatment of drugs and VBL cannot be made at the moment. 

### 5.3. Transjugular Intrahepatic Portosystemic Shunt

For secondary prophylaxis, TIPS was compared to sclerotherapy [120, 165–170] as well as to VBL [119]. In all but two studies [169, 171] patients treated with TIPS had lower rates of recurrent bleeding. Three meta-analyses [172–174] summarized the available studies and found a significant lower probability of rebleeding in the TIPS treated patients. The incidence of hepatic encephalopathy was higher in the TIPS group. A difference in mortality was not evident.

### 5.4. Surgery

Shunt surgery has been shown to be effective in the prophylaxis of rebleeding from esophageal varices. This has been shown for nonselective as well as for selective shunts (e.g., distal splenorenal shunt) comparing operative shunts with no therapy or endoscopic sclerotherapy [129, 175–186]. As in TIPS, the most important side effect was the incidence of hepatic encephalopathy.

One study [187] compared noncovered TIPS with a small diameter prosthetic portacaval H-Shunt. Both shunts led to an adequate reduction in portal pressure, but patency rates of the operative shunts were higher over time. This led to a lower rate of rebleeding as well as to a decrease in mortality in patients with the surgical shunt. A meta-analysis compared different portosystemic shunts (TIPS, diverse surgical shunts) with endoscopic treatment [188]. All shunts were equally effective in reducing the risk of rebleeding. The incidence of hepatic encephalopathy was higher in patients who received a shunt procedure. TIPS was complicated by a high incidence of shunt dysfunction. Comparing the different shunt procedures, there was no difference in survival.

A reasonable approach for secondary prophylaxis (Figure 5) is to perform VBL alone in patients with contraindications for beta blocker therapy or in patients who suffer from side effects of beta blocker therapy. Patients who tolerate drug treatment well should be placed on a combination therapy. If there is more than one episode of rebleeding, TIPS or a surgical shunt should be considered in Child-Pugh A patients. Patients in a good Child-Pugh C or Child-Pugh B state should undergo TIPS placement and might be evaluated for liver transplantation. In patients with a good liver function and refractory ascites, a primary TIPS insertion instead of VBL and/or drug treatment is an attractive option, as not only the risk of rebleeding but also the refractory ascites is treated. Patients with decompensated liver disease who are not suitable for TIPS should undergo evaluation for liver transplantation and repeated endoscopic treatment in combination with beta blocker therapy.

## 6. Gastric Varices 

In contrast to esophageal variceal bleeding, prevention and treatment of bleeding from gastric varices and from portal gastropathy are less well evaluated in controlled clinical studies.

**Figure 6:**
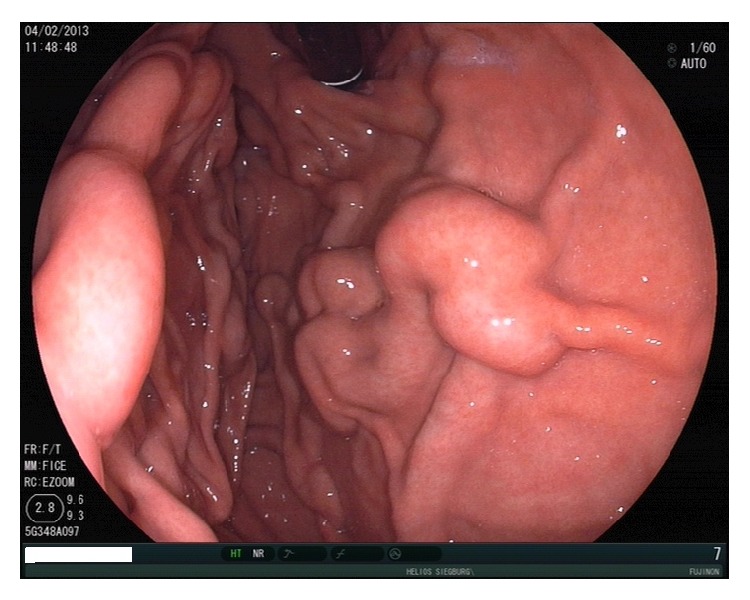
Isolated gastric varices type II located in gastric corpus in a patient with splenic vein thrombosis.

According to Sarin et al. [189] gastric varices are endoscopically classified as gastroesophageal varices type I (lesser curvature), gastroesophageal varices type II (greater curvature), isolated gastric varices type I (located in the gastric fundus), or isolated gastric varices type II (any location in the stomach except for the gastric fundus; Figure 6).

The diagnosis of gastric varices is made by endoscopy. In case of doubt of the diagnosis, endosonography with Doppler sonography allows further differentiation. If only isolated gastric varices are present, the exclusion of portal or splenic vein thrombosis as the underlying cause is mandatory. 

About one-fifth of the patients with portal hypertension develop gastric varices [189]. In patients with gastrointestinal bleeding due to portal hypertension, bleeding from gastric varices is the cause in 5–10% of patients [190]. The risk of the first bleeding from gastric varices is lower than the risk of first bleeding from esophageal varices (4% in one and 9% in three years) [191]. The risk of recurrent bleeding is dependent on the localization of the varix: isolated varices in the gastric fundus (53%) bear the highest risk of recurrent bleeding, followed by varices of the greater curvature (19%) and lesser curvature (6%) [189]. The prophylactic treatment of esophageal varices by VBL does not increase the risk of secondary gastric varices compared to propranolol [192].

Almost no data is available on whether medical treatment for the primary prophylaxis of bleeding from gastric varices is effective. Pathophysiological considerations warrant the use of nonselective beta blockers for this indication [190]. One trial including 27 patients with gastric varices studied the injection of cyanoacrylate for primary prophylaxis of bleeding from large gastric varices and found the injection of cyanoacrylate to be safe and effective in primary prophylaxis [193]. However, before recommending cyanoacrylate injection as prophylactic therapy, more studies are necessary. 

Data for the treatment of acute bleeding from gastric varices is sparse. Therapy with terlipressin or somatostatin is recommended although controlled studies are lacking. The endoscopic treatment of choice is injection with cyanoacrylate [194–196]. Control of bleeding is as high as 90% and more effective than sclerotherapy or band ligation in one trial [197], whereas another study found VBL and cyanoacrylate injection to be equally effective in terms of control of acute bleeding but reported higher rebleeding rates in the VBL group [198]. Known complications of cyanoacrylate injection include mucosal ulcerations as well as thromboembolism. TIPS insertion is highly effective with control of bleeding in more than 90% of patients [199, 200] and should be considered in patients in whom endoscopic therapy fails.

The use of nonselective beta blockers and nitrates for prophylaxis of rebleeding was shown in one study to be ineffective [201]. The comparison of cyanoacrylate with propranolol for secondary prophylaxis has shown no difference between the two treatment modalities in terms of rebleeding or mortality but found more complications in the cyanoacrylate group [202]. Another study compared TIPS with cyanoacrylate in patients with bleeding from gastric varices. TIPS was shown to be more effective for prevention of recurrent bleeding, with no difference in mortality [203]. These results are in contrast to a retrospective analysis that found TIPS and cyanoacrylate to be equally effective in controlling and preventing gastric variceal hemorrhage with no significant differences in survival [204]. Patients who received TIPS experienced significantly more long-term morbidity [204]. Nevertheless, the above-mentioned studies have to be interpreted with caution, since they included patients with different types of gastric varices. 

## 7. Portal Hypertensive Gastropathy

**Figure 7:**
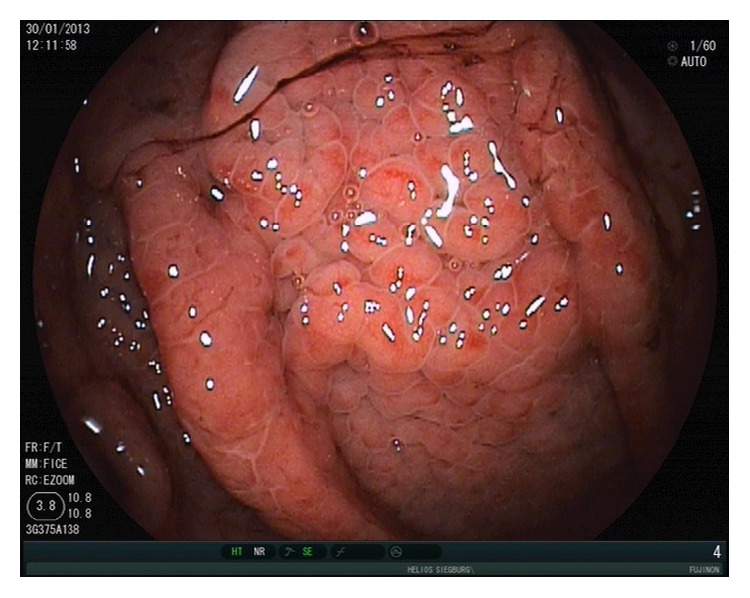
Portal hypertensive gastropathy with the typical “snakeskin” pattern in a patient with liver cirrhosis.

**Figure 8:**
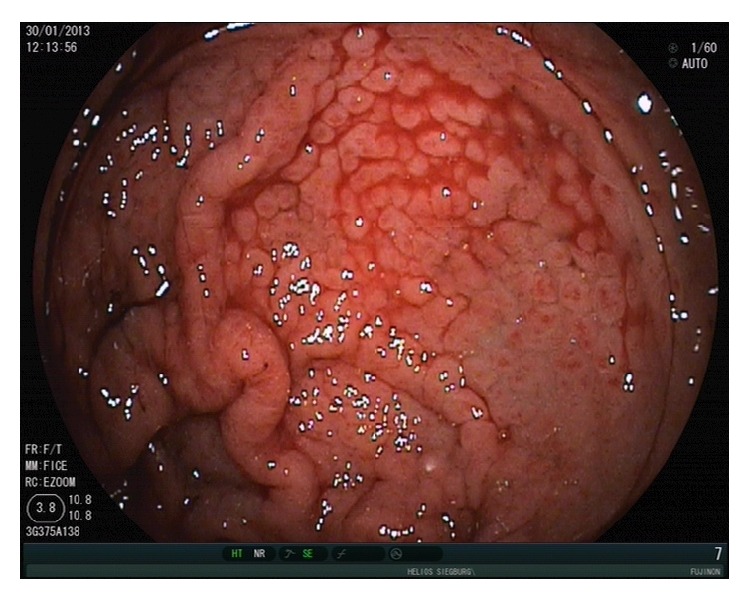
Acute bleeding from portal hypertensive gastropathy in a patient with liver cirrhosis.

The diagnosis of portal hypertensive gastropathy is made by endoscopy. Typical signs are mosaic, also called “snakeskin” pattern of erythema (Figure 7). These signs are mostly found not only in the fundus and body but also in the gastric antrum [205, 206]. More severe forms present with red punctuate erythema, diffuse hemorrhagic lesions, and/or brown spots that indicate submucosal hemorrhage [207]. Several attempts to classify portal hypertensive gastropathy have been made, with the classification of the NIEC (North Italian Endoscopic Club for the study and treatment of esophageal varices) being widely accepted [208–210]. The classification system of the NIEC is a two-category system (mild or severe) and describes two types of lesions: the mosaic-like pattern as a marker of mild disease and red marks as a marker of severe gastropathy [210]. Histopathological features of portal hypertensive gastropathy are vascular ectasia of the mucosal and submucosal veins and capillaries [207]. The exact pathogenesis of portal hypertensive gastropathy is unknown. Important factors in the pathogenesis are the presence of portal hypertension as well as hyperemia of the gastric mucosa. The incidence of portal hypertensive gastropathy in patients with liver cirrhosis who are screened for varices is around 30 to 45% at the end of the follow-up period [207]. One study found portal hypertensive gastropathy more common in patients with gastroesophageal varices than in patients with esophageal varices alone [211]. Several authors assumed that the endoscopic treatment of esophageal varices aggravates portal hypertensive gastropathy [211]. The worsening is often transient, and portal hypertensive gastropathy shows regression in more than 40% of patients after VBL [212]. Acute bleeding from portal hypertensive gastropathy (Figure 8) is a rare event, with an incidence of less than 3% in three years [213]. One study that evaluated the cause of GI bleeding in 1496 patients found bleeding from portal hypertensive gastropathy to be the cause in 0.8% of patients, accounting for 8% of nonvariceal bleeding in patients with liver disease [214]. The probability of chronic bleeding is around 10–15% in three years [13].

There is only one small trial that studied the effect of nonselective beta blockers on portal hypertensive gastropathy [215]. Twenty-four patients with nonbleeding portal hypertensive gastropathy received 160 mg propranolol per day in a double-blind placebo controlled cross-over trial. Endoscopic grading of portal hypertensive gastropathy improved after propranolol in nine patients compared to three after placebo [215]. 

The therapy of acute bleeding from portal hypertensive gastropathy is mainly based on drugs that decrease portal pressure. In one study, 14 portal hypertensive patients with heavy diffuse bleeding from portal hypertensive gastropathy received propranolol in a dose of 24 to 480 mg per day. Within three days, bleeding ceased in 13 (93%) of patients [215]. Since the study did not have a control group of untreated patients, the results have to be interpreted with caution. A small study compared octreotide, vasopressin, and omeprazole for therapy of acute bleeding. In this setting, octreotide was more effective than omeprazole or vasopressin [216]. Terlipressin was also shown to be effective in acute bleeding from portal hypertensive gastropathy [217].

No studies that investigated the role of endoscopic treatment using argon-plasma coagulation in acute or recurrent bleeding from portal hypertensive gastropathy are available. If medical therapy fails, TIPS insertion or surgical shunt is an option [13, 218, 219].

In the secondary prophylaxis of bleeding from portal hypertensive gastropathy, one study including 54 patients showed that propranolol is effective in the prevention of rebleeding [220]. In the group of the propranolol treated patients 65% were free of rebleeding after one year compared to 38% in the control group. After 30 months of follow-up, 52% of the patients in the propranolol group were free of rebleeding compared to 7% of the untreated patients [220]. 

In summary, the risk of bleeding from portal hypertensive gastropathy is low, and primary prophylaxis is therefore not necessary. In patients with recurrent bleeding from portal hypertensive gastropathy, propranolol should be considered for secondary prophylaxis. 

## 8. Ectopic Varices

In the majority of cases, portal hypertension as a complication of liver cirrhosis or an extrahepatic obliteration of the portal vein is the cause for the development of ectopic varices. Gastroesophageal varices are the most common portosystemic collaterals that develop in patients with portal hypertension. In comparison, ectopic varices are less often found. In one case series including 1218 patients with varices due to portal hypertension, 43 (3.5%) patients had ectopic varices [221]. They are predominantly located in the duodenum, jejunum, ileum, colon, and the rectum [222]. Other sides like the urinary bladder or gallbladder bed have been described. 

By definition, ectopic varices are dilated portovenous vessels of the gastrointestinal mucosa that are located outside the esophagus or the stomach. They have their origin from preexisting small veins of the gastrointestinal mucosa that are portosystemic collaterals between the portal vein and the inferior vena cava. Under normal conditions, the small size and the high vascular resistance in these vascular beds restrain blood flow through the collaterals [223]. In portal hypertension, the deep mucosal veins of the GI tract become enlarged [224], and the number and size of the vessels are increased [225].

**Figure 9:**
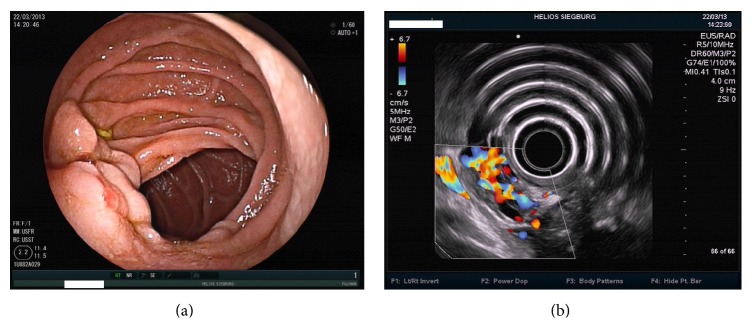
Duodenal varices in a patient with portal vein and splenic vein thrombosis: (a) endoscopic picture, (b) endosonographic picture.

A large retrospective analysis of 5664 endoscopic procedures revealed 13 patients with duodenal varices. Sixty-nine percent of these patients had also esophageal varices [226]. More than 80% of duodenal varices are found in the descending part of the duodenum (Figure 9); duodenal varices in the transverse part (14%) and in the duodenal bulb (3.5%) [227] are less common.

The reported prevalence of varices of the jejunum and ileum is in the range of 1.9% to 8.7% in patients who were examined because of obscure GI bleeding using either capsule endoscopy or enteroscopy [228, 229]. However, in patients with portal hypertension small bowel varices were found in 69% of patients [230]. In patients with liver cirrhosis and anemia, capsule endoscopy demonstrated small bowel varices in 16% to 26% of patients [231, 232]. These findings were substantiated by a study that found varices of terminal ileum in 18% of cirrhotic patients with portal hypertension who were systematically examined during ileocolonoscopy [233].

Varices and angiodysplasia of the colon in patients with portal hypertension are termed portal hypertensive colopathy [234]. Colonic varices are found in 34% to 46% of patients with portal hypertension [235, 236].

Rectal varices are defined as variceal veins that extend more than 4 cm above the anal border and, in contrast to hemorrhoids, do not prolapse into the proctoscope on examination [237]. Rectal varices are a frequent complication of portal hypertension and liver cirrhosis. They are found in 89% and 56% of patients with portal hypertension without or with liver cirrhosis, respectively [238].

Stomal varices are a common complication in patients with surgical stoma and chronic liver disease. The reported prevalence is between 27% and 31% of patients [239, 240].

**Figure 10:**
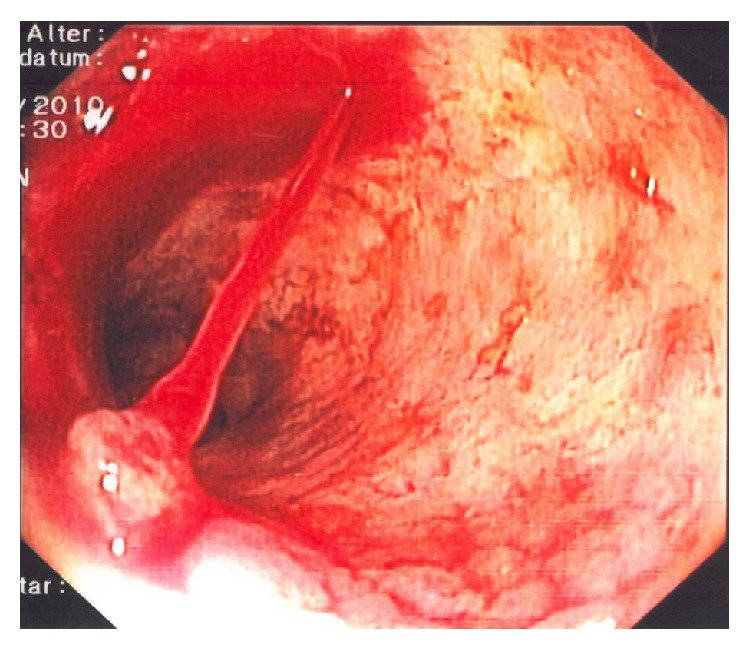
Acute bleeding from a rectal varix in a patient with liver cirrhosis.

Bleeding from ectopic varices (Figure 10) is a rare event. It accounts for 1–5% of all gastrointestinal bleeding episodes in patients with portal hypertension [221, 241]. Endoscopy is the most important diagnostic tool. In patients with portal hypertension, acute bleeding, and negative findings on upper endoscopy, bleeding from ectopic varices has to be considered. In these patients, accurate examination of the duodenum is mandatory. Examination of the jejunum makes double-balloon enteroscopy or capsule endoscopy necessary. 

Colonoscopy is the principal method for the diagnosis of colonic varices. One study found rectal varices via endoscopy in 43% and via EUS in 75% of patients with portal hypertension, pointing out that rectal varices might be overlooked by conventional endoscopy [235].

In patients in whom bleeding from ectopic varices is suspected but endoscopy was negative, NMR with NMR angiography is the diagnostic tool of choice and allows the identification of ectopic varices in most patients.

### 8.1. Therapy

There are no randomized trials that compared the different treatment modalities that are available for the therapy of bleeding from ectopic varices. In patients with liver disease and portal hypertension, it seems reasonable to extrapolate the supportive treatment guidelines of acute esophageal variceal hemorrhage (cautious transfusion regimens, vasoactive drugs, and antibiotics) to patients with bleeding from ectopic varices.

#### 8.1.1. Sclerotherapy/Injection Therapy

Endoscopic therapy of ectopic varices is mainly based on sclerotherapy or injection therapy. Controlled studies for which method is the best are not available, but case reports showed that both sclerotherapy with aethoxysklerol and injection of the varix with cyanoacrylate are feasible [242–247].

Band ligation may be useful for temporary hemostasis of bleeding duodenal varices [235, 248], but rebleeding is a problem with ligation therapy. Additional treatment following band ligation for duodenal varices is therefore mandatory. Furthermore, it might be technically difficult to obtain visualization of the bleeding side in the duodenum with the banding device in place. The choice of endoscopic therapy is therefore mainly based on personal expertise and location of the bleeding site.

#### 8.1.2. Surgery and TIPS

Portacaval shunts are effective therapy measures in patients with uncontrolled or recurrent bleeding from ectopic varices [186, 249, 250]. Another option in patients without portal vein thrombosis is TIPS insertion. Several case reports that show that TIPS is an effective option in the treatment of ectopic varices have been published [251–254]. As in patients with bleeding from esophageal varices, caution is necessary in patients with decompensated liver disease and/or hepatic encephalopathy.

#### 8.1.3. Interventional Radiology

Angiography is not only a diagnostic tool but allows therapeutic interventions. Angiography of ectopic varices is performed by either a direct access of the portal system through transhepatic portography or indirect by visualization of the venous phase after mesenteric or splenic arteriography. Ectopic varices are displayed as atypical splanchnic vessels that are fed from the superior or inferior mesenteric vein showing a hepatofugal flow [255].

Balloon-occluded retrograde transvenous obliteration (B-RTO) was successfully performed for patients with duodenal varices [256, 257]. B-RTO can obliterate not only varices but also the afferent and efferent veins and should be considered for treating duodenal varices. 

#### 8.1.4. Medical Therapy

From a pathophysiological point of view the application of beta blockers does make sense in patients with ectopic varices, but no data from controlled trials that investigate the role of nonselective beta blockers and/or nitrates are available.

## 9. Conclusions

Bleeding from varices is a common and often life-threatening complication of portal hypertension. The best modalities for prophylaxis and treatment of acute bleeding from esophageal varices were investigated in numerous clinical studies, and evidence based clinical guidelines are available. Only few studies investigated the prophylaxis and treatment of bleeding from gastric varices, and almost no controlled studies investigating prophylaxis and treatment of bleeding from portal hypertensive gastropathy and from ectopic varices have been conducted. 

A trained interdisciplinary team consisting of endoscopists, hepatologists, and specialized nurses as well as interventional radiologist if appropriate should administer treatment of acute bleeding related to portal hypertension. Treatment is done according to the current guidelines. Endoscopic therapy is a key aspect, but pharmacologic treatment with vasopressors and antibiotic treatment are also important components of successful patient care.
